# Are smokers protected against SARS-CoV-2 infection (COVID-19)? The origins of the myth

**DOI:** 10.1038/s41533-021-00223-1

**Published:** 2021-02-26

**Authors:** Naomi A. van Westen-Lagerweij, Eline Meijer, Elisabeth G. Meeuwsen, Niels H. Chavannes, Marc C. Willemsen, Esther A. Croes

**Affiliations:** 1 The Netherlands Expertise Centre for Tobacco Control, Trimbos Institute, Utrecht, The Netherlands; 2 Department of Health Promotion, Maastricht University, Maastricht, The Netherlands; 3 Public Health and Primary Care, Leiden University Medical Center, Leiden, The Netherlands

**Keywords:** Health care, Diseases

## Abstract

A number of recent studies have found low percentages of smokers among COVID-19 patients, causing scientists to conclude that smokers may be protected against SARS-CoV-2 infection. National and international media were interested in this story and we soon began receiving questions about this topic in general practice. In this article, we shed light on the process that resulted in the misinterpretation of observational research by scientists and the media. We also point out the methodological flaws of various studies on which hasty conclusions were based. Finally, we address the role of primary healthcare providers in mitigating the consequences of erroneous claims about a protective effect of smoking.

Recently, a number of observational studies found an inverse relationship between smoking and severe acute respiratory syndrome coronavirus 2 (SARS-CoV-2) infection (coronavirus disease 2019 (COVID-19)), leading to a (social) media hype and confusion among scientists and to some extent the medical community. The finding that smoking is not associated with SARS-CoV-2 infection contradicts earlier studies which found that smokers are more vulnerable to infections in general and to respiratory infections in particular. Smoking is known to increase the risk of infection of both bacterial and viral diseases, such as the common cold, influenza and tuberculosis^1^, and smoking is a putative risk factor for Middle East respiratory syndrome coronavirus infection^2^. Could it be possible that SARS-CoV-2 is the big exception to the rule? To date, there is no strong evidence (i.e., evidence based on causal research) that smokers are protected against SARS-CoV-2 infection. Moreover, there is growing evidence that smokers have worse outcomes after contracting the virus than non-smokers^3^.

If there is no strong evidence that smokers are protected against SARS-CoV-2 infection, how is it possible that such a potentially dangerous claim gained so much attention? Due to the great need for knowledge about COVID-19 and the associated ‘publication pressure’, several manuscripts were quickly published in peer-reviewed journals without undergoing adequate peer review. Also, many manuscripts did not initially follow the traditional time-consuming peer review process but were immediately shared online as a preprint. Although scientific discussions could be continued afterwards on the preprint servers, the media and many scientists did not follow these discussions. As a result, studies designed to report correlations within a non-causal framework were quickly picked up via (social) media and presented within a causal framework. We now know that <20% of COVID-19 preprints actually received comments^4^. Also, <50% of the COVID-19 preprints uploaded in the first few months of the pandemic (January–April) have been published in peer-reviewed journals so far^5^. Both findings emphasise the great caution needed in interpreting (social) media claims of preprint results.

It seems the tobacco industry benefited from the (social) media hype, since exposure to claims about a protective effect of smoking was associated with an increase in tobacco consumption among Chinese citizens during the pandemic^6^. Also in other countries, an increase in tobacco consumption among smokers has been reported^7,8^, possibly influenced by this hype. In France, researchers first suggested that nicotine may play a role in protecting smokers^9^, triggering a run on nicotine products among the general public. Interestingly, the lead author of this research has been funded by the tobacco industry in the past, and also other researchers who have made similar claims can be linked with the tobacco industry, indicating a possible conflict of interest. According to the Global Center for Good Governance in Tobacco Control, the tobacco industry was actively involved in downplaying the role of smoking in COVID-19 by spreading claims that smoking or vaping protects against COVID-19^10^.

So, what research was this claim based on in the first place? In the early months of the COVID-19 pandemic, most studies describing the relationship between smoking and COVID-19 were based on Chinese patient groups^11–18^. These studies, in which smoking status was not a primary exposure of interest, were subsequently brought together in several systematic reviews and meta-analyses^19–25^. Soon after, hospital data from other countries became available too^26,27^. Overall, the findings suggested that smokers were underrepresented among COVID-19 patients based on the prevalence of smoking in the general population. The studies, however, made comparisons without adjusting for a number of factors that are associated with smoking status, such as age, gender, socio-economic status, ethnicity and occupation. The studies also contained other major methodological flaws, including incompleteness of data (the majority of the studies had >20% missing data on smoking status^3^), selection bias^28^ and misclassification bias^3^. Here we use two examples (one Chinese and one French study) to illustrate the most common problems with these studies.

1. Guan et al. is one of the largest Chinese studies on smoking and COVID-19, with data on 1590 patients from 575 hospitals across China^11^. Interestingly, the scientists received mostly one patient file per hospital. It is unclear on what grounds these patients were selected for inclusion in the study. Furthermore, 93% of all patients were categorised as: ‘smoking status: never/unknown’^11^. According to a peer reviewer of a different study, ‘unknown’ can be explained by the fact that many patients were too ill to answer the questions about smoking^29^. When we look more closely at specific patient groups in the data, we see that, of the 24 included chronic obstructive pulmonary disorder (COPD) patients, only 3 had ever smoked (12.5%); the other 21 patients are found in the category ‘smoking status never/unknown’^11^. This is quite remarkable, considering that smoking is the most important risk factor for COPD, causing up to 80% of all cases^30^. Guan et al. also found an unusually low number of smokers among patients with a cardiovascular or cerebrovascular disease^11^.
2. A university hospital in Paris appears to have collected their data more systematically: they asked 482 COVID-19 patients whether they smoked or had done so in the past, resulting in only 9 missing answers^27^. They reported only 5% of current daily smokers in their patient group. But what was left out of the (media) attention was that 32% of patients reported being former smokers, defined as ‘anyone having smoked in the past, occasionally or daily, and had abstained from smoking prior to COVID-19 onset’^27^. This definition allows individuals to have been a smoker the day before development of COVID-19 symptoms. There were more serious limitations of this study: a relatively small patient group recruited in an affluent neighbourhood with many hospital staff among the patients; exclusion of the most critical cases of COVID-19 (i.e. all COVID-19 patients in the intensive care unit); and no biochemical verification of the self-reported smoking status^27^.

Aside from the methodological issues in these studies, there are more reasons why hospital data are not suitable for determining the risk of SARS-CoV-2 infection among smokers. First, many critically ill COVID-19 patients have severe comorbidities that may exclude them from being admitted to a hospital or intensive care unit. This may, for example, apply to patients with serious cardiovascular and lung diseases, which are often the result of long-term smoking. Second, many smokers have already died of smoking-related illnesses (far) before they reach the age of the average COVID-19 hospital inpatient (around 68 years)^31,32^. And the final and most important reason is that hospital data are collected cross-sectionally (i.e. determining risk factor and disease at the same time). In epidemiology, cross-sectional studies are the weakest form of observational studies. The highest achievable outcome in cross-sectional research is to find a correlation, not causation. Only cohort studies of sufficient size, in which a group of patients is followed over a longer period of time, would be able to determine whether smokers are actually protected against SARS-CoV-2 infection or not.

In the meantime, it is imperative that any myths about smoking and COVID-19 among the general public are expelled, especially considering the growing evidence that smokers have worse outcomes once infected^3^. There is no easy solution to the spread of health misinformation through social media, but primary healthcare providers (HCPs) can play an important role in mitigating its harmful effects. What are some practical steps primary HCPs can take? First, in line with national guidelines, primary HCPs can choose to ask patients about their smoking status during consultations, inform smokers about the dangers of smoking, advise smokers to quit smoking and offer cessation support to all smokers. As face-to-face cessation support may now be limited, primary HCPs can point out the availability of support at a distance, such as telephone quitlines or eHealth interventions. Second, primary HCPs can inform patients about the harmful relationship between smoking, COVID-19 and other serious illnesses, for example, by addressing the issue on their website or on posters/television screens in the waiting room. We encourage HCPs to use the information provided by recognised international organisations, such as the World Health Organisation. Third, since exposure to health misinformation on social media is more common among youth and young adults^6^, primary HCPs may choose to actively bring up the subject of smoking and COVID-19 in consultations with youth and young adults and advise non-smokers to never start smoking.

A HCP’s advice for smoking cessation has always been very important, but in these COVID-19 times it is more urgent than ever before.
